# Microbiota biodiversity in inflammatory bowel disease

**DOI:** 10.1186/1824-7288-40-32

**Published:** 2014-03-31

**Authors:** Donatella Comito, Antonio Cascio, Claudio Romano

**Affiliations:** 1 Department of Pediatric Sciences, University of Messina, Messina, Italy; 2 Department of Human Pathology, University of Messina, Messina, Italy

**Keywords:** Dysbiosis, Eubiosis, Inflammatory bowel disease, Crohn’s disease, Ulcerative colitis

## Abstract

Gut microbiota plays a significant role in human health and energy balance, and provides protection against disease states. An altered balance between microbiota and its host (dysbiosis) would appear to contribute to the development of Inflammatory Bowel Disease (IBD), Crohn’s Disease (CD) and Ulcerative Colitis (UC). CD and UC are chronic inflammatory diseases of the gastrointestinal tes.

## Introduction

The human gut is sterile at birth but immediately after and during infancy, it is colonized by numerous types of microorganisms with temporal and inter-individual variations, which can be influenced by environmental factors [1].

The adult human intestinal microbiota contains around 10^14^ bacterial cells and up to an estimated 1,000 different bacterial species [2], resident at any time in the gastrointestinal tract. Bacterial numbers range from very low levels in the stomach to high levels of luminal contents in the human colon. There are four predominant species: *Firmicutes*, *Bacteroides*, *Proteobacteria* and *Actinobacteria*. The most abundant bacteria phyla found in the healthy human large intestine are Gram-negative Bacteroidetes and Gram-positive low-GC Firmicutes [3].

The gut microbiota performs a number of critical roles in the adult: metabolism of dietary components and cholesterol, enterohepatic cycling of bile acids, vitamin synthesis, intestinal motility, immune system modulation [4].

Preserving eubiosis is important for maintaining the integrity of the intestinal epithelium [5] and contributing to antimicrobial defenses. Intestinal epithelial cells (IECs) provide a physical barrier between luminal microbes and underlying intestinal tissues to control defense and tolerance [6]. IECs express pattern recognition receptors, they can recognize microbial pathogen-associated molecular patterns and respond to intestinal microbes through secretion of cytokines and antimicrobial proteins. Furthermore they can up-regulate surface molecules [7]. The presence of IgA reduces intestinal proinflammatory signals and drives diversity in gut microbiota [8]. A defective antibacterial, genetically driven barrier allows translocation and regulation of the microbiota. Commensal bacteria can have an anti-inflammatory effect on the developing immune system; for example, in the neonatal period T helper type 2 response is predominant [9]. Host–microbe interaction is a two-way dialogue; in healthy conditions, balanced mechanisms regulate the host’s immunological tolerance to continuous stimulus of resident gut microbiota and their metabolic end products. Human nutritional components and dietary patterns cleary impact microbial composition and function of intestine. Long- term differences in diet seem to affect gut microbiota, as digestion of diet with fiber and plants component, without demonstrated role in the pathogenesis of IBD [10].

### Dysbiosis in IBD: cause or effect of the disease?

Dysbiosis is defined as an abnormal ratio of beneficial and aggressive bacterial species. A more recent study suggests that it is a key characteristic of IBD, although it remains unclear whether dysbiosis is a cause or a consequence of the mucosal inflammation [11].

Recent metagenomic studies have analyzed microbial compositions in IBD, suggesting that not only is the quantity of commensal bacteria reduced but also the quality and composition of the microbiota are altered with reduction of Firmicutes and Bacteroidetes. It has been reported that in IBD patients compared to healthy controls there is an abnormal colonization of the ileal mucosa by Adherent/Invasive *E. coli* (AIEC) [12] and reduced ileal mucosal concentrations of *Faecalibacterium prausnitzii*, member of *Clostridium* subsets IV. This condition can be respectively associated to severity of ileal disease and predictive of high risk for early reactivation of ileal Crohn’s disease [13]. Recently, a molecular subset of *Bacteroides fragilis*, termed enterotoxigenic *B. fragilis* has been identified in abnormal concentrations in patients with active IBD [14].

**Figure 1:**
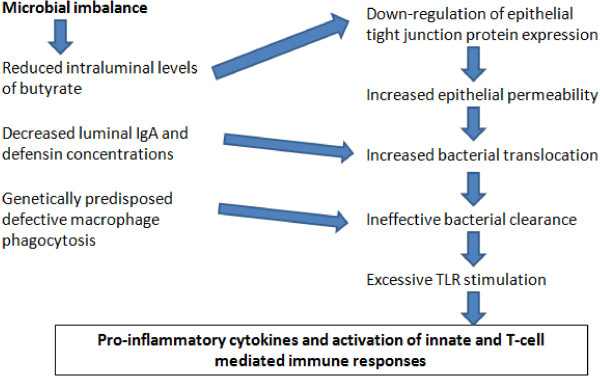
Dysbiosis and intestinal inflammation.

There is also a different microbiota composition between inflamed and non-inflamed mucosa [10] with loss of tolerance and defectiveness in the production or function of anti-bacterial peptides, such as defensins by Paneth cells. It has been demonstrated that alpha-defensin production is reduced in ileal CD [15], while antibacterial peptide expression is reduced in colonic CD [16]. These alterations cause loss of tolerance to commensal flora, and to amplification and maintenance of inflammatory response to intestinal pathogens. In trying to establish a pathogenic role of dysbiosis in IBD, microbial imbalance may be a trigger for a range of mechanisms determining low intraluminal levels of butyrate, down-regulation of epithelial tight junction protein expression and at least increased epithelial permeability. Epithelial barrier dysfunction brings about increased bacterial translocation through the lamina propria, which is worsened by decreased luminal IgA and defensin concentrations (Figure 1) [17]. Killing of bacteria reaching the lamina propria, through the “leaky” epithelium, is also impaired by a genetically predisposed defective macrophage phagocytosis. Ineffective bacterial clearance leads to excessive TLR stimulation, secretion of pro-inflammatory cytokines and activation of innate and T-cell mediated immune responses. This disrupted mechanism of tolerance in epithelial cells may recognize dysbiosis as a *primum movens* of induction of intestinal inflammation by commensal bacteria and pathogens.

One agent that has raised a great deal of controversy is *Mycobacterium avium* subspecies *paratuberculosis* (MAP), agent of chronic granulomatous enterocolitis in ruminants, has been considered a possible etiologic agent of CD [18]. Prevalence studies of MAP in CD patients from many countries worldwide have reported widely divergent results ranging from 0% to 100% [19]. The possible role of MAP has also been supported by the identification of MAP DNA in media inoculated with peripheral blood mononuclear cells (PBMC) from patients with CD [20]. Kirkwood et al. found evidences of MAP infection in 45% of children with CD, 35% of children with UC, and 11% of non-IBD children. These results support the hypothesis that MAP infection of intestinal tissue, perhaps associated with blood-borne spread, may be implicated especially in the pathogenesis of pediatric CD [21]. However, the failure of triple antimycobacterial therapy has questioned whether this pathogen is involved in the pathogenesis of CD [22].

One other enteric bacterial pathogen that has been considered in the pathogenesis of IBD is *Clostridium difficile*. Toxin A of *C. difficile* may have the ability to reactivate IBD [23]. In a Polish retrospective study, Mir et al. have examined the rate of *C. difficile* infection (CDI) in 123 new pediatric IBD patients and overall prevalence was 8.1%, significantly lower than in Poland but much higher than in the general population [24]. Other studies have reported a low incidence of CDI in pediatric IBD [25,26], but all studies support testing for *C. difficile* in suspected cases of new onset or flare pediatric IBD, and new biomarkers have been identified to distinguish *C. difficile* colonization from diseases that need therapy [26].

## Genoma and microbioma in IBD

The insights obtained during genoma wide association studies have shed new light on the interaction of bacteria with the mucosal immune system and the pathways by which intestinal microbiota may contribute to chronic mucosal inflammation. Loss of function mutations in NOD2 are responsible for dysregulation of ileal microflora and of hypo-production of antimicrobial peptides, such as defensins, and may increase disease susceptibility by altering interactions between ileal microbiota and mucosal immunity [27]. CD patients with *NOD2* gene defects have an impaired ability to recognize and process bacterial products, and this may lead to an inappropriately long immune response. In 2006, Duerr et al. [17] showed that association of variants of the interleukin IL-23 receptor gene with both CD and UC confers strong protection against CD. Some CD patients have variants of the ATG16L1 and Immunity-related GTPase family M Protein (IRGM) autophagy genes, implying a defective capacity to process cell degradation products as well as bacteria, and to eliminate pro-inflammatory stimuli [28].

Three exclusive theories have been proposed concerning the implication of bacteria in the etiopathogenesis of IBD: the first is related to an involvement of persistent pathogen, the second, an abnormally permeable mucosal barrier leading to excessive bacterial translocation, and the third, a breakdown in the balance between putative “protective” as against “harmful” intestinal bacteria which can promote inflammation.

The alteration of the balance of the microbiota by host tissue due to environmental factors on the microbiota can be considered as the basis of disease. Interestingly, a similar reduction in bacterial diversity has recently been noted in the oral microbiota of children with CD, but not in those with UC or healthy children. Only adult studies examining biopsies from both active and quiescent UC suggest a reduction in bacterial diversity. Early-onset IBD (onset <6 years of age) and very early onset IBD (onset <2 years of age) present a unique opportunity to study the impact of immunological status, neutrophil role, gut microflora, metabolic pathways, and environmental factors on genetic predisposition and the natural history of IBD. Disease phenotypes are primarily colonic with frequent perianal and oral localization, and with a more rapid and progressive disease course. Colectomy has been required in a high percentage of children. It may be associated with immunodeficiency states, neutrophil defect, metabolic disease and monogenic defects (IL-10) [29].

### Implications for therapy

Dysbiosis can be considered an important pathogenetic factor in IBD. Manipulation of the microbiota may be a new therapeutic approach to IBD.

#### Probiotics and prebiotics

Probiotics have been described as “live microbial supplements that beneficially affect the consumer by improving intestinal microbial balance” [30]. The mechanisms of action of the probiotics can be quite disparate. It has been shown that they modulate the permeability of epithelial barriers, alter the inflammatory potential of epithelial cells, compete with pathogens for mucosal colonization, or directly modify the activity of immune cells [31]. Recently, IL-17–producing T-helper cells (Th17 cells) have been regarded as crucial for the control of several chronic inflammatory diseases such as IBD. Th17 cells are abundant in the intestine, and the development of these cells seems to be regulated by dendritic cells and macrophages in the lamina propria [32]. Thus, orally administered probiotics (such as *L. rhamnosus GG*, a strong inducer of TGF-beta, IL1-beta and IL-23) could affect the development of Th17 cells and ameliorate clinical symptoms [33].

Numerous small studies reported on the efficacy of probiotics in pouchitis. Gosselink et al. [34] showed that first episodes of pouchitis were observed significantly less frequently in UC patients who received *Lactobacillus rhamnosus GG* daily versus placebo (7% vs 29%). *L. rhamnosus GG* has been demonstrated to inhibit TNF-α -mediated apoptosis of intestinal epithelial cells [35].

A small open label trial in 2008 showed that maintenance therapy for 34 children in remission from ulcerative colitis (UC), with probiotic *Escherichia coli* Nissle 1917 (EcN) is as effective as mesalazine [36], confirming the data of adult UC [37]. EcN has been demonstrated to have pro-apoptotic effects on pro-inflammatory γδ-T cells, which are increased in the blood of IBD patients [38,39].

VSL#3, a mixture of eight bacterial species, has been associated to maintenance of remission pouchitis [40] and antibiotic-induced remission in UC. A pediatric trial demonstrated significant efficacy of VSL#3 in the induction and maintenance of remission in pediatric active UC. Twenty-nine consecutive patients (mean age, 9.8 years) with newly diagnosed UC were randomized to receive either VSL#3 or an identical placebo associated to concomitant steroid induction treatment and oral mesalazine maintenance treatment. Patients treated with VSL#3 and concomitant conventional therapy had a significantly higher rate of remission compared with the group treated only with conventional therapy, with a significantly lower incidence of relapse within one-year follow-up. There were no biochemical or clinical adverse events related to VSL#3 [41].

Although mixtures of probiotic species have shown some efficacy in treating IBD [42], they are not considered effective for intestinal homeostasis and can populate the intestine only transiently. Overall, no consistent benefits have been observed in CD patients.

A randomized placebo-controlled study provides evidence that inflammation of the rectal mucosa in 31 children with active distal UC can be reduced by local administration of *Lactobacillus reuteri* ATCC55730 in addition to standard oral mesalazine. Compared with the placebo-treated group, the probiotic-treated group had reduced Mayo scores (clinical and endoscopic features) and histological scores. In this group, concentrations of mucosal pro-inflammatory cytokines (IL-1b, TNF-α and IL-8) decreased significantly, whereas anti-inflammatory cytokine (IL-10) increased significantly [43].

Diet can influence the composition of the microbiota. Dietary manipulation with prebiotics, non-digestible food ingredients that stimulate the growth and/or activity of bacteria in the digestive system, can promote the growth of commensal bacteria (lactobacilli and bifidobacteria) [44]. However, clinical trials are lacking in IBD patients, because of high dropout rates due to troublesome side effects, such as increased gas and bloating.

Nutritional therapy can improve the inflammatory status of CD by restoring the composition of the mucosal microbiome. Recently, a case of CD gut microbiome dysbiosis that responded to nutritional therapy has been described [45]. However, these data need to be confirmed by multicenter studies and possible clinical trials.

#### Antibiotics

Antibiotics may have clinical efficacy in a subgroup of IBD patients to targeted killing/stasis of bacteria. The most used are amoxicillin, tetracycline, metronidazole, neomycin or rifaximin. Clinical efficacy has been shown in pouchitis. A meta-analysis in 2011 supported a role of antibiotics in induction of remission of CD, UC and perianal fistulae [46]. Significant variability exists in antibiotic use for children hospitalized with IBD exacerbation, and further studies are needed to determine the optimal antibiotic therapy for this condition [47]. Levine and Turner [48], in a retrospective analysis, have demonstrated that an 8 weeks course of azithromycin and metronidazole may be effective in inducing clinical remission in mild-moderate luminal CD in children and young adults. However, prolonged administration of antibiotics can be accompanied by systemic adverse effects and can induce CDI. Rifaximin, a polymer with negligible intestinal absorption, has been shown to modulate the colonic microbiota of patients with CD by increasing the concentration of anti-inflammatory commensal, such as *Bifidobacteria* and *Faecalibacterium prausnitzii.* Furthermore, in a preliminary experience in pediatric IBD, rifaximin appeared to be effective and well tolerated [49].

#### Fecal microbiota transplantation

Fecal microbiota therapy (FMT) is a treatment that involves transplanting the intestinal bacteria of a healthy person to a person with the disease [50]. In literature, the first use of FMT is described for treating pseudomembranous colitis, refractory to antibiotic therapy, with good results [51]. Rubin et al has reported 79% remission after nasogastrically delivered fecal transplantation in a cohort of 74 patients with recurring CDI [52]. After FMT, the modification of the composition of the microbiota can be seen with an increase in the presence of the *Bacteroidetes* and *Firmicutes* species [53]. Several randomized placebo-controlled clinical trials have confirmed that FMT is safe and effective for treating *C. difficile* infection [54,55], and a recent eradication of CDI in a UC patient was described [56]. Nine cases of IBD flares (8 UC and 1 CD) successfully treated with FMT in IBD, without side effects have been reported [57-60]. Further studies are needed to understand what components are most responsible for efficacy, and to clarify if it is possible to develop an “artificial stool”, eliminating theoretical infectious risks and unpleasantness [61].

#### Others

Other proposed therapeutic strategies include: blocking attachment of adherent bacteria by several probiotic bacteria or bacterial components (such as fibers and emulsifiers) [62] and enhancing defective bacterial killing in genetically susceptible hosts by Granulocyte-macrophage colony-stimulating factor [63].

## Conclusions and perspectives

An increased understanding of the molecular mechanisms that modulate the innate immune response to bacteria and antigens in the intestine is important to better define the pathogenesis of IBD. However, it is not yet clear whether dysbiosis contributes to the development of IBD or is instead a consequence of the disease. Antibiotic therapy has not been demonstrated to be effective in the treatment of IBD, except in specific circumstances. FMT as a treatment for IBD holds therapeutic potential and deserves further investigation.

## Methods

Any experimental research reported in the review have been performed with the approval of an appropriate ethics committee and carried in compliance with the Helsinki Declaration or internationally recognized guidelines.

## Competing interests

Authors declare that they don’t have financial and/or non financial competing interest, in this article.

## Authors’ contributions

DC, AC & CC participated in literature search, data analysis, manuscript writing. In addition, CC submitted the manuscript. All authors revised and approved the manuscript.
